# Patients with rare diseases: from therapeutic orphans to pioneers of personalized treatments

**DOI:** 10.15252/emmm.201708365

**Published:** 2017-11-27

**Authors:** Christoph Klein, William A Gahl

**Affiliations:** ^1^ Department of Pediatrics Dr. von Hauner Children's Hospital University Hospital LMU Munich Munich Germany; ^2^ Undiagnosed Diseases Program and National Human Genome Research Institute National Institutes of Health Bethesda MD USA

**Keywords:** Genetics, Gene Therapy & Genetic Disease

## Abstract

Common diseases, such as cancer, diabetes mellitus, or Alzheimer's disease, affect a large segment of the population, which justifies the enormous financial allocations for translational and clinical research. In contrast, the ~5,000 known rare disorders affect only very few patients each, even though the cumulative disease burden is substantial. This influences not only the general appreciation of research to address rare diseases, but also the allocation of research funds. Importantly, however, studying rare diseases has contributed enormously to our understanding of human biochemistry, cell and developmental biology, and physiology. For example, Linus Pauling and Vernon Ingram's discovery of a structural difference and amino acid variant in the beta‐globin protein, which causes monogenic hemoglobinopathies such as sickle cell disease or thalassemia, issued in the era of molecular medicine (Pauling *et al*, 1949). Subsequently, numerous genetic defects in critical genes controlling differentiation and/or function of cells and organs have been identified and opened new possibilities for molecular diagnosis.

*“Nature is nowhere accustomed*

*more openly to display her secret mysteries*

*than in cases where she shows traces of her*

*workings apart from the beaten path;*

*nor is there any better way to advance the*

*proper practice of medicine than to give*

*our minds to the discovery of the usual law of*

*nature, by the careful investigation of*

*cases of rarer forms of disease*.

*For it has been found in almost all things, that*

*what they contain of useful or of applicable,*

*is hardly perceived unless we are deprived of*

*them, or they become deranged in some way.”*

William Harvey, 1657 Letter to Dr. John Vlackveld

From a therapeutic perspective, research on rare diseases has also set the stage for crucial advancements in medicine. Patients with rare primary immunodeficiency diseases were the first to be cured by transplantation of allogeneic blood stem cells (Bach *et al*, 1968; Gatti *et al*, 1968). The first successful gene therapy studies were also performed in patients with rare diseases, such as severe combined immunodeficiency syndromes (Cavazzana‐Calvo *et al*, 2000), factor IX deficiency (Nathwani *et al*, 2011), and rare genetic diseases of the retina (Bainbridge *et al*, 2008). These pioneering initiatives paved the way for incorporating new techniques into our medical armamentarium and thereby created new therapeutic options for many other diseases.

Rare disease research is now fulfilling the promise of targeted therapy, that is, personalized medicine. This begins with one or more patients who are affected with a particular disorder the genetic basis of which requires elucidation. The first step involves careful medical evaluation, and our clinical tools are now sufficiently sophisticated: laboratory and imaging studies, tissue histology, and so on, all provide clues for diagnosis. And yet, hundreds of extremely rare illnesses languish without a recognized genetic basis. One critical issue relates to which signs and symptoms are part of the disease spectrum and which ones are not; here, additional cases can greatly contribute to a diagnosis. Fortunately, the searching of phenotypic databases is aided by the use of computer‐based ontologies that employ common terms to describe physical findings; examples include Phenotips and Human Phenotype Ontology (Box 1) (Kohler *et al*, 2014).

**Box 1: Platforms and initiatives**

**Platforms**

Human Gene Ontology–http://www.geneontology.org/

Phenotips–https://phenotips.org/

Matchmaker–http://www.matchmakerexchange.org/

**Initiatives**

Global Genes–https://globalgenes.org/

NIH Undiagnosed Diseases Program–https://www.genome.gov/27544402/the-undiagnosed-diseases-program/

International Rare Diseases Research Consortium (IRDIRC)–http://www.irdirc.org/

The Genetic and Rare Diseases Information Center (GARD)–https://rarediseases.info.nih.gov/

Care‐for‐Rare Foundation–http://www.care-for-rare.org/en

DAAD thematic network “Research for Rare Diseases and Personalised Medicine”–https://www.ten-for-rare.com/

European Reference Network “Rare immunodeficiency, autoinflammatory and autoimmune diseases” (ERN RITA)–http://rita.ern-net.eu/

Research for Rare–http://www.research4rare.de/

The second major challenge in rare disease research is associating a molecular diagnosis with the documented phenotype. In this regard, the annotated human genome sequence along with next‐generation sequencing has proven revolutionary. Single nucleotide polymorphism analysis detects copy number variants (deletions and duplications) and runs of homozygosity, which allows bioinformaticians to focus their search for disease‐causing genes. High‐throughput sequencing quickly and easily provides the 60 million and 3.2 billion bases, respectively, of a patient's genome or exome. With appropriate coverage and the help of continuously improving computational algorithms, novel and potential disease‐causing variants that differ from reference sequences can be identified. Software programs prioritize these variants based upon segregation using Mendelian inheritance models, variant frequency, estimates of deleteriousness, and so on. These “filters” reduce the number of candidate gene variants from tens of thousands to a few.

Still, uncertainty reigns, because so many genetic complexities are far from being understood. Current challenges include questions regarding the functional relevance of rare genetic variants, the importance of mutations in non‐coding regions, the influence of epigenetic mechanisms in rare diseases, and the role of somatic mutations and mosaicism in specific disorders.

Despite these impediments, the number of genes associated with rare phenotypes continues to increase rapidly. Two principles are now considered axiomatic in the field. The first one is the value of sharing data. Clearly, patients with a rare disease want to share their stories, both to foster research and to create a community. They are willing to sacrifice some measure of privacy when their life is at stake, and they prove it by putting their stories on social media. Institutional Review Boards are coming to realize this, and now allow more sharing of personal information, with fully informed consent. Rare disease researchers also understand the importance of sharing data in order to link genes to phenotypes, and physicians connect with basic scientists to diagnose and treat rare and new human diseases. Computer databases for phenotype and genotype analyses provide the substrate for interactions, and new global initiatives catalyze collaborations.

The second principle addresses the importance of functional studies to link a specific phenotype to a potentially disease‐causing gene. Such investigations can help to elucidate new pathways, identify potential drug targets, and lead to new therapies. These projects, based on a phenotype, the patient's DNA and cells, and animal models, have piqued the interest of pharmaceutical companies, who have recognized the potential applications to common diseases.

Specifically, pathomechanisms of single‐gene disorders are less complex than those of common disorders, so therapeutic targets can be more readily detected by investigating the genetic causes. Examples of pharmaceutical interventions include enzyme replacement therapies for metabolic diseases, antisense oligonucleotides for spinal muscular atrophy, or small molecules for the treatment of cystic fibrosis and myasthenia. Examples of cell and gene‐based therapies include the intramuscular gene therapy of lipoprotein lipase deficiency and hematopoietic stem cell gene therapy for adenosine deaminase deficiency. In fact, the discovery of therapeutic targets by rare disease research is not only the basis for studies that aim to cure these patients, but provides the inspiration to target pathognomonic “Achilles heels” in more common disorders. When Ogdon Bruton first described a patient with recurrent episodes of bacterial pneumonia associated with deficient serum gammaglobulins (Bruton, 1952), he laid the foundation for therapeutic administration of immunoglobulins and targeted therapies for B‐cell lymphomas.

While rare diseases provide an inspiration for personalized therapeutics at all levels, major issues and challenges persist. Randomized, double‐blinded clinical trials, the gold standard of therapeutic research, are not feasible for very rare diseases; novel models for statistical analyses are needed. Incentives for pharmaceutical companies are minimal, although the US Orphan Drug Act and the European Medical Agency have bolstered drug approvals for “orphan diseases”. There are extensive regulatory hurdles and costs associated with clinical studies. In addition, the ethical aspects involving principles of distributive justice and equal access are often daunting.

Nevertheless, the prospect for advancing the treatment of rare diseases has never been greater. In the USA, the NIH Common Fund has devoted more than US$200 million to create the NIH Undiagnosed Diseases Network (UDN) that includes seven clinical sites, a coordinating center, two sequencing cores, a model organisms core, and a metabolomics core. The UDN arose from the Undiagnosed Diseases Program (UDP), established within the Intramural Research Program of the NIH in 2008 to help diagnose patients with a long “diagnosis odyssey” and to discover new diseases and insights into human biochemistry, physiology, and cell biology (Box 1). Since 2008, the UDP has reviewed more than 4,000 applications, evaluated more than 1,100 patients during a 1‐week admission to the NIH Clinical Center, made more than 200 diagnoses of extremely rare diseases, and discovered new diseases (St. Hilaire *et al*, 2011). The UDP also created a unique database, called UDPICS, that contains genetic information and searchable phenotypes using the Phenotips ontology, all based upon comprehensive clinical investigations and exome sequencing of patients and their families.

Following on the success of the UDP and the UDN, the Undiagnosed Diseases Network International (UDNI) has been established to foster access to rapid/correct diagnoses and therapies throughout the world. The UDNI has annual meetings, a charter, policies for data sharing, and arrangements for using an international database. Moreover, UDPs are now being initiated throughout the world (Baynam *et al*, 2017).

Other international alliances and networks have been formed and have proven strong drivers of change (Box 1). In 2009, the Care‐for‐Rare Foundation was established in Germany which follows the vision that no child should be destined to die of a rare disease, regardless of national or ethnic origin and the parents’ financial opportunities. The award‐winning philanthropic foundation supports research in a global alliance of clinicians and scientists (care‐for‐rare alliance), education (care‐for‐rare academy), global outreach to lay and professional communities (care‐for‐rare awareness) and individual patients (care‐for‐rare aid), and a competitive award program (care‐for‐rare awards). In addition, international organizations such as REACT, IRDIRC, and ERN continue to help patients with rare and undiagnosed diseases, and pharmaceutical companies have taken notice: witness the development of ivacaftor by the Cystic Fibrosis Foundation and Vertex Pharmaceuticals (Wainwright *et al*, 2015).

Finally, let us recall a time when another disorder, childhood leukemia, was universally fatal. Some physician‐scientists, including Sydney Farber in Boston and Emil Frei and Emil Freireich at NIH, refused to accept certain death as the destiny of these children. Despite great opposition from their colleagues, they introduced the concept of anti‐leukemia chemotherapy and thus produced the very first cures (Mukherjee, 2010). Nowadays, four of five children with leukemia can be cured. This has generated a huge wave of philanthropic support, which has transformed the world. Today, we face the same challenge to save the lives of children and adults with other rare, life‐threatening diseases that cannot yet be cured. To achieve this goal, funding from government and non‐profit agencies is as critical as support from private institutions. Rare disease research has proven to be transformational; now, it is making individualized medicine a reality.

## Conflict of interest

The authors declare that they have no conflict of interest.
